# Cascading (3D) reconstruction procedure of composite structures from microtomography data

**DOI:** 10.1016/j.mex.2023.102177

**Published:** 2023-04-09

**Authors:** Marouane Kabbej, Valérie Guillard, Hélène Angellier-Coussy, Valentin Thoury-Monbrun, Nathalie Gontard, Laurent Orgéas, Sabine Rolland du Roscoat, Sébastien Gaucel

**Affiliations:** a IATE, CIRAD, INRAE, Université Montpellier, Institut Agro, Montpellier 34060France; b CNRS, 3SR Lab, Université Grenoble Alpes, UMR 5521, Grenoble F-38000, France

**Keywords:** *Cascading (3D) reconstruction procedure of composite material*, Particle morphology, (3D) Structure building, Heterogeneous size distribution, (3D) tri-phasic structure

## Abstract

Reconstruction of three-dimensional (3D) structure from experimental image acquisition (e.g., from micro computed tomography data) is very useful in composite material science. Composite considered are characterized by a dispersion of particles in a continuous phase. Many properties of the composite (e.g., mass transfer properties) depend on its structural assembly. A reliable prediction of these properties requires to well represent this structure and especially, the region at the vicinity of the dispersed phase. (3D) structure generation must thus permit to (1) simplify the real composite structure observed to make it compatible with further modelling tasks (e.g., meshing constraints in finite elements methods, computation time) and (2) keep enough representativeness of the structure of the specimen to produce reliable numerical predictions. This article describes an innovative, cascading (3D) reconstruction procedure of composite material from microtomography data.

• First step of this pipeline is the extraction of relevant structural markers from microtomography images using image analysis.
• Second step is the modelling of the distribution of the structural markers selected (statistical laws).
• Third and final step is the reconstruction of the (3D) structures based on the pre-determined distribution laws in a RVE (representative volume element) of the composite.

## Graphical abstract

**Figure:**
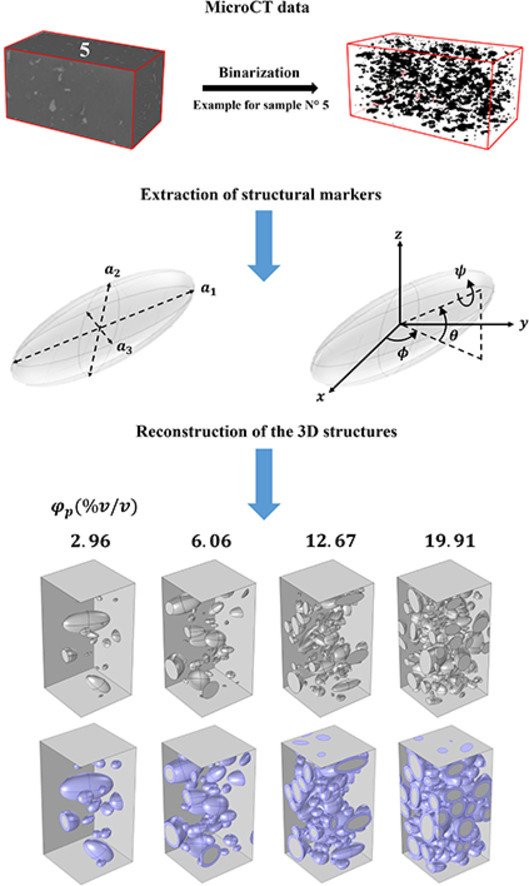

Specifications table

**Table:** 

Subject Area:	Materials Science

More specific subject area:	*(3D) Numerical modelling*
Method name:	*Cascading (3D) reconstruction procedure of composite material*
Name and reference of original method:	*The procedure is an original one and combined different standard/published techniques and novel ones. The standard/published techniques are:* *- Tschopp MATLAB code*[7] *- (3D) Objects Counter'' in ImageJ software*[4] *- MorphoLibJ plugin in ImageJ software*[2] *- Conversion expressions of Tait-Bryan angles into Euler angles*[1]
Resource availability:	*Link to dataverse:* *Size distribution of cellulose particles into PP/cellulose particles* https://doi.org/10.57745/PDQNR6 *Descriptors of the cellulose particles detected in the (3D) microtomography images of composites PP/cellulose* https://doi.org/10.57745/KXPWXK

## Method details

Step 1. (3D) microtomography images analysis

**Fig. 1:**
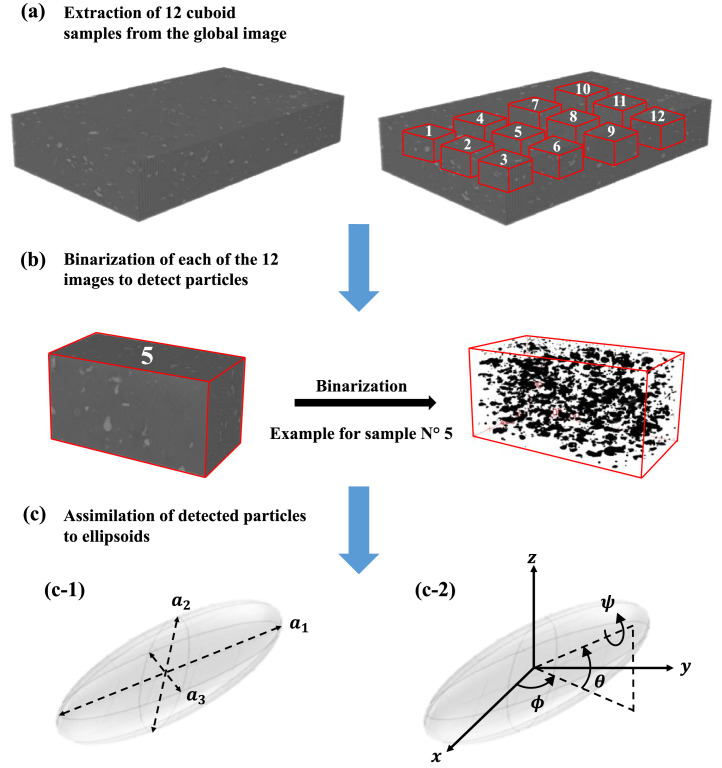
Images analysis steps of the (3D) microtomography: (a) Extraction of 12 cuboid samples from the global image of dimension of 1844 × 1403 × 298 µm^3^;(b) Binarization of each of the 12 images of dimension reported in Table S1.1 of suppl. material. Here is an example of binarization of the sample N°5. In the binary image, the matrix and the particles are in white and black colour respectively; (c) Modelling a particle as an ellipsoid in (3D) space. (c-1) a_1_: largest axis, a_2_: medium axis, a_3_: smallest axis (c-2) ψ: roll angle, θ: elevation angle, ϕ: azimuth angle. All descriptors obtained from the 12 images analysed are available at https://doi.org/10.57745/KXPWXK.

For this work, we used (3D) grayscale tomographic image of the specimen analysed. Tomographic images used were obtained using X-ray microtomography on the ID19 beamline at the ESRF in Grenoble with a resolution of 0.65 µm^3^/voxel. From one (3D) tomographic image of real dimension of 1844 × 1403 × 298 µm^3^ (length x depth x height), 12 cuboid samples of same height, 250 µm, were extracted from different zones (Fig. 1a) to be further analysed (precise dimensions of the twelve samples are given in Table S1.1 of suppl. material).

The analysis time of the global image is considerable (several hours or even days), this is the reason we have chosen to analyse 12 cuboid samples of the global image that reduce considerably the computation time (about 10–15 min for each sample: about 3 h for all the 12 samples). Note that images analysis was performed in a DELL computer with Intel Xeon E-2176 M Processor (2.7 GHz) and 32 Gb of Ram.

Using the option “(3D) Objects Counter'' in ImageJ software [4], segmentation (binarization) was first applied to these 12 cuboid samples by changing grayscale images to binary images: 0 for the matrix and 1 for the fillers (Fig. 1b). This allows the detection and identification of particles. Afterwards, observed particles were assimilated to ellipsoids (Fig. 1c) whose shape descriptors were measured using MorphoLibJ plugin in ImageJ software [2,3].

The distribution of these descriptors is the basis of the generated (3D) structures in this work.

**Fig. 2:**
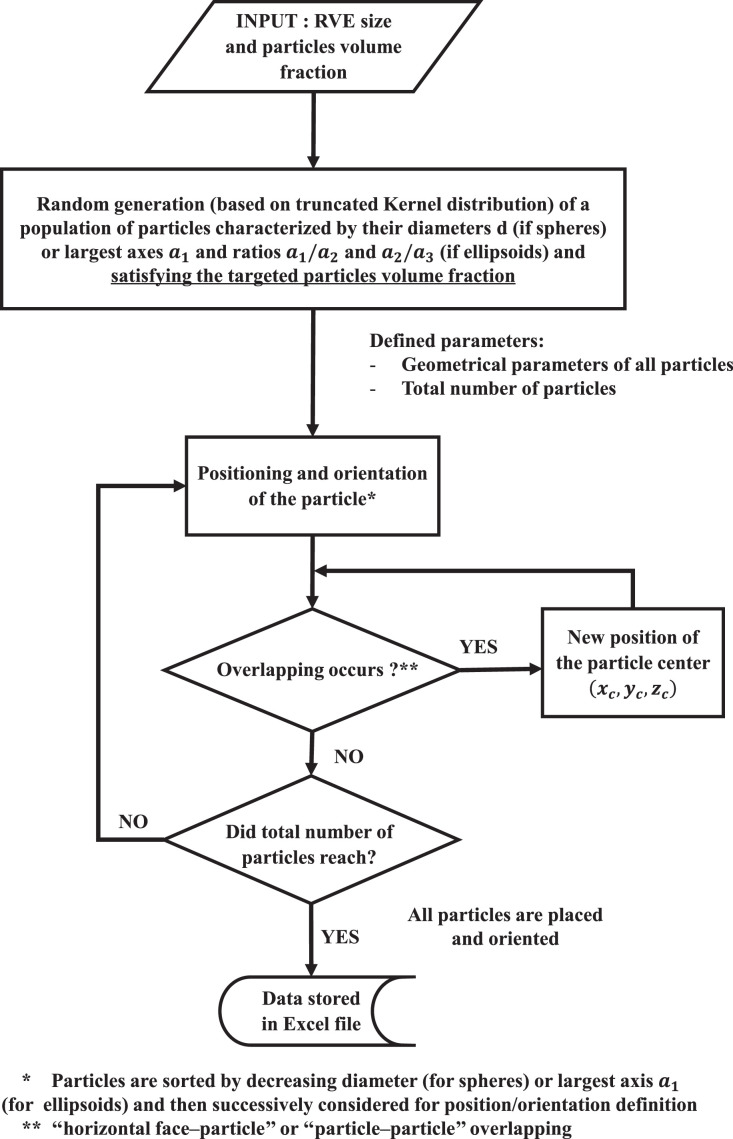
Numerical process to generate the (3D) microstructure of the RVE performed in MATLAB.

In the present work, the particles are considered as ellipsoids and are characterised by morphological parameters, the three axes a_1_, a_2_ and a_3_ (a_1_>a_2_>a_3_) (Fig. 1c-1) and orientation in the composite material, given by three orientation angles (ψ, θ, ϕ) (Fig. 1c-2) which are further detailed.

Step 2. Structural parameters distribution laws

Distribution of descriptors identified in step 1 is then modelled by using a statistical distribution law. The best fit obtained by the statistical tools in Matlab library permits to select the appropriate distribution law.

Step 3. (3D) Structure generation

The MATLAB code developed for generating our (3D) structures was based on Tschopp MATLAB code [7]. The code generates (3D) microstructures composed of a population of non-overlapping ellipsoidal particles heterogeneously distributed in size and orientation, within a periodic RVE.

Organisational chart summarising the structure of the generation algorithm could be found in Fig. 2. Bi-phasic or tri-phasic systems (i.e., obtained by adding a third compartment surrounding the dispersed particles) can be generated with this generation procedure.

### Bi-phasic system

The bi-phasic composite structure is generated in a cuboid shaped representative volume element (RVE) defined by x,y,z ∈[0, L_x_] × [0, Ly] × [0, L_z_], where *z* is the direction orthogonal to the permeation flux for a composite membrane. The RVE is supposed periodic along its vertical faces, to represent an infinite repetitive structure along x and y axis.

Structure generation requires the RVE size and the target particles volume fraction $\varphi_{p,target}$ in% volume/volume (%v/v) as inputs.

The geometric parameters of a population of particles, a_1_, a_2_ and a_3_, are randomly generated using distribution law determined in step 2 and satisfying the target volume fraction of particles, using the criterion

$$\left|\varphi_{p,target}-\varphi_{p}\right|\leq 0.01\%v/v.$$

**Fig. 3:**
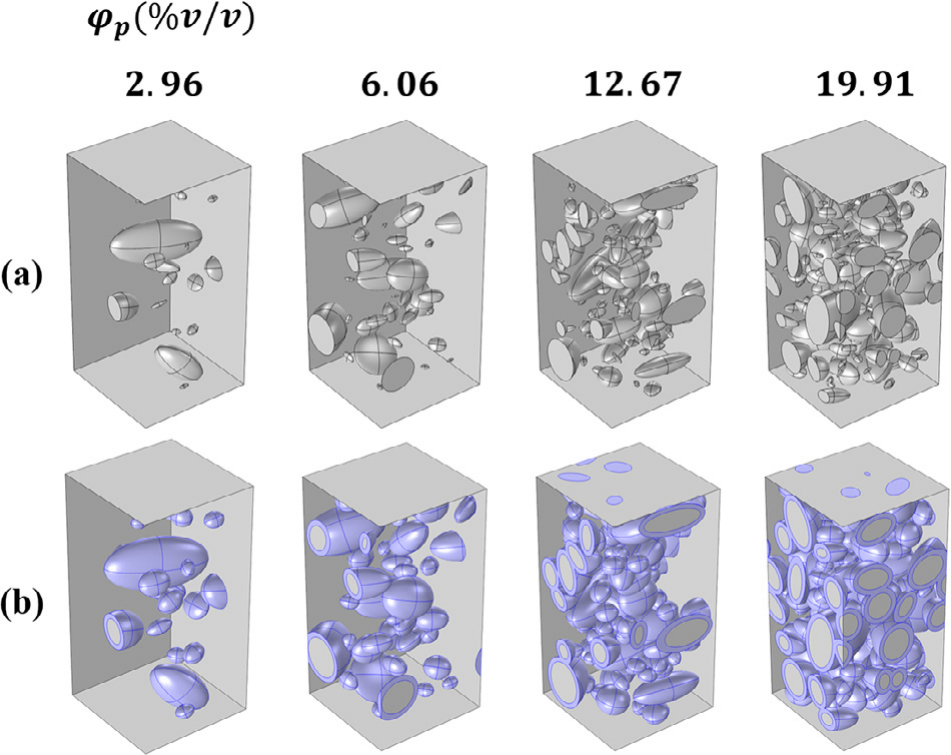
Representation of the RVE showing structures with size-heterogeneous ellipsoidal particles: (a) bi-phasic structures and (b) tri-phasic structures of interphase thickness of 2 µm (purple colour), both generated using the algorithm proposed in that work. The structures are generated for particles volume fraction corresponding to true composite materials ($\varphi_{p}=2.96-6.06-12.67-19.91\%v/v$).

Then, these particles are sequentially positioned and orientated in the periodic RVE following the sequential approach described below:

**i.** The particle's position (i.e. centre coordinates $x_{c},y_{c},z_{c}$) is randomly generated using uniform distribution. Note that the ellipsoidal particles are sorted by decreasing largest axis a_1_, large particles being positioned first. At the same time, the orientation angles, i.e. roll ψ, elevation θ and azimuth ϕ angles (Fig. 1c-2), are randomly generated using the appropriate distribution identified in step 2.
**ii.** The non-overlapping of the particle with the horizontal faces of the RVE (z=0 and $z=\;L_{z}$) and with the existing particles is tested. If the non-overlapping tests are successful, the particle is added to the structure and then the next particle is considered for steps i–ii. If at least one of the non-overlapping conditions is not satisfied, then a new position is drawn, orientation being unchanged, and step ii is performed again on the updated particle. This last sequence is repeated until the particle is added to the structure. It should be noted that if a particle intercepts one of the vertical faces of the RVE then, the particle section outside from the RVE is shifted to the opposite face, in order to ensure the periodicity of the RVE (Fig. 3a).

### Tri-phasic system

The tri-phasic composite structure is built by considering an interphase of fixed thickness around each particle of the already generated bi-phasic structures. The interphase volume is thus different for each particle keeping constant the initial particle volume. For the interphase compartment, the periodicity at vertical faces of the RVE still applies. For particles close to the upper (resp lower) face of the RVE, the interphase may intercept the boundary. In that case, the intercept with the exterior of the RVE is removed (Fig. 3b).

## Method validation

**Step 1.** To test the cascading (3D) reconstruction procedure proposed here, a composite of cellulose (= the particles or filler), dispersed into Polypropylene (= the matrix) with a filler volume fraction of $\varphi_{p}=2.96\%v/v$ was considered ([5]a). The (3D) tomographic image of dimension of 1844 × 1403 × 298 µm^3^ was transformed into 12 cores cuboid samples cumulating a total of 21,570 particles (Fig. 1a) (see S1 in Supp Material).

To detect the particles, different thresholding values of the segmentation (binarization) were tested in all the 12 cores of cuboid samples. To validate it, volume fraction of the particles on the binarized cuboids were calculated and compared to the experimental one. Globally, the more the thresholding increases, the more the volume fraction of the particles decreases. In the present case, the thresholding of 105 was selected as it permits to achieve a calculated volume fraction very close to the experimental one, $\varphi_{p}=2.96\%v/v$.

Before analysing the 12 cores of cuboid samples to extract the geometrical descriptors, the particles intercepting the edges of the cores were removed. Also, the particles with less than 30 voxels were rejected, to avoid meshing errors in the upcoming numerical simulations, reducing the total number of particles from 21,570 to 12,812. These rejected particles represent 40.60% in number and 0.47% in volume of the 21,570 particles, respectively. Despite their large number, these tiny, rejected particles represent less than 1% of the volume of all the particles, which is low. Theoretically, these tiny particles may be fragments of cellulose which, due to their large number, generate a large volume of interphase. However, no such particles were visible in 2D images of cellulose particles as observed and described in [6].

Finally, geometrical descriptors of the 12,812 remaining particles were measured using MorphoLibJ plugin in ImageJ software (see Section 6.2 of the ref. [2]) assimilating particles to ellipsoids (Fig. 1c). The ellipsoids of inertia, determined by the MorphoLibJ plugin are characterised by the following nine coefficients,

• Coordinates of the centroid $\left(x_{c},y_{c},z_{c}\right)$
• Axes $a_{1}^{{}^{\prime }},\;a_{2}^{{}^{\prime }},\;a_{3}^{{}^{\prime }}$, corresponding to the length of the largest, medium and smallest axis, respectively $\left(a_{1}^{{}^{\prime }}>a_{2}^{{}^{\prime }}>a_{3}^{{}^{\prime }}\right)$
• Tait-Bryan angles (ψ,θ,ϕ)for orientation with x-y-z rotation convention (extrinsic rotations)

Ellipsoid of inertia has the same first-order and second-order moments than the studied real particle i.e. same centre of inertia and orientation but not the same volume. To preserve the real volume, the axes obtained with the MorphoLibJ plugin $\left(a_{1}{}^{\prime },a_{2}{}^{\prime },a_{3}{}^{\prime }\right)$ were normalised and converted as follows:

(1) $$a_{i}=a_{i}^{\prime }{\left(\frac{V_{real}}{V_{ell}}\right)}^{\frac{1}{3}}for\;i\in \left\{1,2,3\right\}$$

where $V_{real}$ and $V_{ell}$ are the volume of the real particle and the ellipsoid of inertia $\left(V_{ell}=\pi /6\;\times {a_{1}}^{\prime }\;\times {a_{2}}^{\prime }\;\times {a_{3}}^{\prime }\right)$, respectively.

MorphoLibJ provides Tait-Bryan angles (ψ,θ,ϕ) with x-y-z extrinsic rotation convention. It corresponds to a succession of three rotations about the **space-fixed principal axes**, namely the x, y and z axes. The first rotation is by the roll angle ψ about *x*-axis, the second is by the elevation angle θ about *y*-axis and the third is by the azimuth angle ϕ about *z*-axis.

The variation intervals of the angles ψ,θ and ϕ considered by the MorphoLibJ plugin are [−180°,180°], [−90°,90°] and [−180°,180°] respectively. With these intervals of angles, we encounter cases where particles are orientated in a similar way but with different combinations of the triplet (ψ,θ,ϕ). Then, we used some properties of symmetry to reduce the variation intervals of both the angles ψ and ϕ to [0°,180°], so that each particles orientation corresponds to a single combination of the triplet (ψ,θ,ϕ).

For the upcoming numerical simulations, it is necessary to convert the Tait-Bryan angles (ψ,θ,ϕ) obtained from the MorphoLibJ plugin of ImageJ to the classical Euler angles (γ,β,α) with z-x-z extrinsic rotation convention that COMSOL software uses as orientation inputs of particles. The used convention of the Euler angles corresponds to a succession of three rotations about the **space-fixed principal axes** (extrinsic rotations), namely the x and z axes (z-x-z rotation convention). The first rotation is by intrinsic angle γ about the z-axis, the second is by nutation angle β about the x-axis and the third is by precession angle α about the z-axis. The conversion expressions are detailed in the section S2 of suppl. material (see also Table 1 in page 19 of the reference of [1]).

The experimental particles sizes $\left(a_{1},a_{2},a_{3}\right)$ found from particles’ analysis were a_1_=11.57 ± 9.87, a_2_=6.83 ± 4.77 and a_3_=4.35 ± 2.43 µm (mean values of all particles).

**Step 2**. To randomly generate particle descriptors selected in the previous step (i.e., sizes and orientation angles), their distribution must be first modelled. In the current case study, a non-parametric Kernel distribution model (proposed by MATLAB) was used to generate descriptors’ distribution as it was identified as the best law to describe the experimental distribution for the selected descriptors.

Kernel distribution is a non-parametric representation of the probability density function (PDF) of a random variable x. A kernel distribution is defined by a smoothing function K(x) and a bandwidth value h, which control the smoothness of the resulting density curve:

(2) $$f\left(x\right)=\frac{1}{N\cdot h}\sum_{i=1}^{N}K\left(\frac{x-x_{i}}{h}\right)$$

where *x_1_*, *x_2_*, …, *x_n_* are random samples from an unknown distribution, *N* is the sample size, *K(x)* is the kernel smoothing function, and *h* is the bandwidth. *K* is often chosen as the density of a standard Gaussian function (mean µ=0;   Std. dev.  σ=1):

(3) $$K\left(x\right)=\frac{1}{2\sqrt{\pi }}e^{-\frac{1}{2}x^{2}}$$

**Fig. 4:**
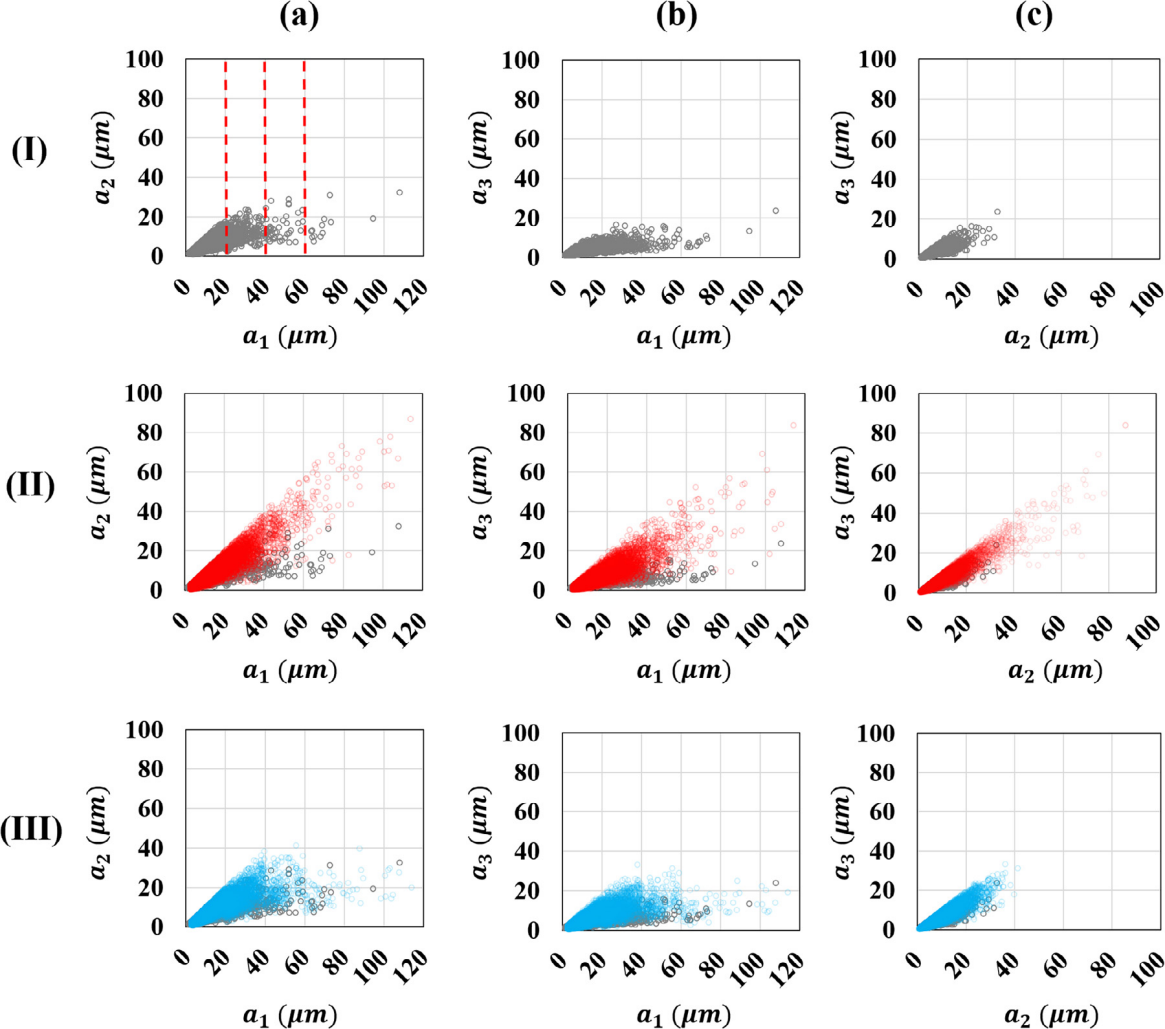
Scatter plots of axes a_1>_a_2>_a_3_ of ellipsoid-shaped particles from three different sources: (grey bullets) values from 12,812 particles analysed in a X-ray tomographic image, (red bullets) values from 15,000 generated particles considering a single Kernel distribution for the ratio a1/a2, (blue bullets) values from 15,000 generated particles considering four Kernel distributions for the ratio a1/a2, defined in four intervals for a1 described by dashed red lines in I-a. The correlations between a_1_ and a_2_ (column a), a_1_ and a_3_ (column b) and a_2_ and a_3_ (column c) are presented. Raw data available at https://doi.org/10.57745/PDQNR6.

The experimental distribution of the largest axis a_1_, the axes ratios a_1_/a_2_ and a_2_/a_3_ were fitted using the truncated Kernel law. The use of triplet (a_1_, a_1_/a_2_, a_2_/a_3_), instead of direct fitting of (a_1_, a_2_, a_3_), was motivated by the dependency observed between axis in data from image analysis. To preserve the correlation between a_1_, a_2_ and a_3_ on the whole a_1_ value range it was necessary to use four Kernel distributions for a_1_/a_2_ defined in four separate zones of a_1_ (zones bounded by red dashed lines in Fig. 4-Ia): a_1_≤ 20 µm; 20 〈 a_1_ ≤ 40 µm; 40 < a_1_ ≤ 60 µm and a_1_ 〉 60 µm.

**Fig. 5:**
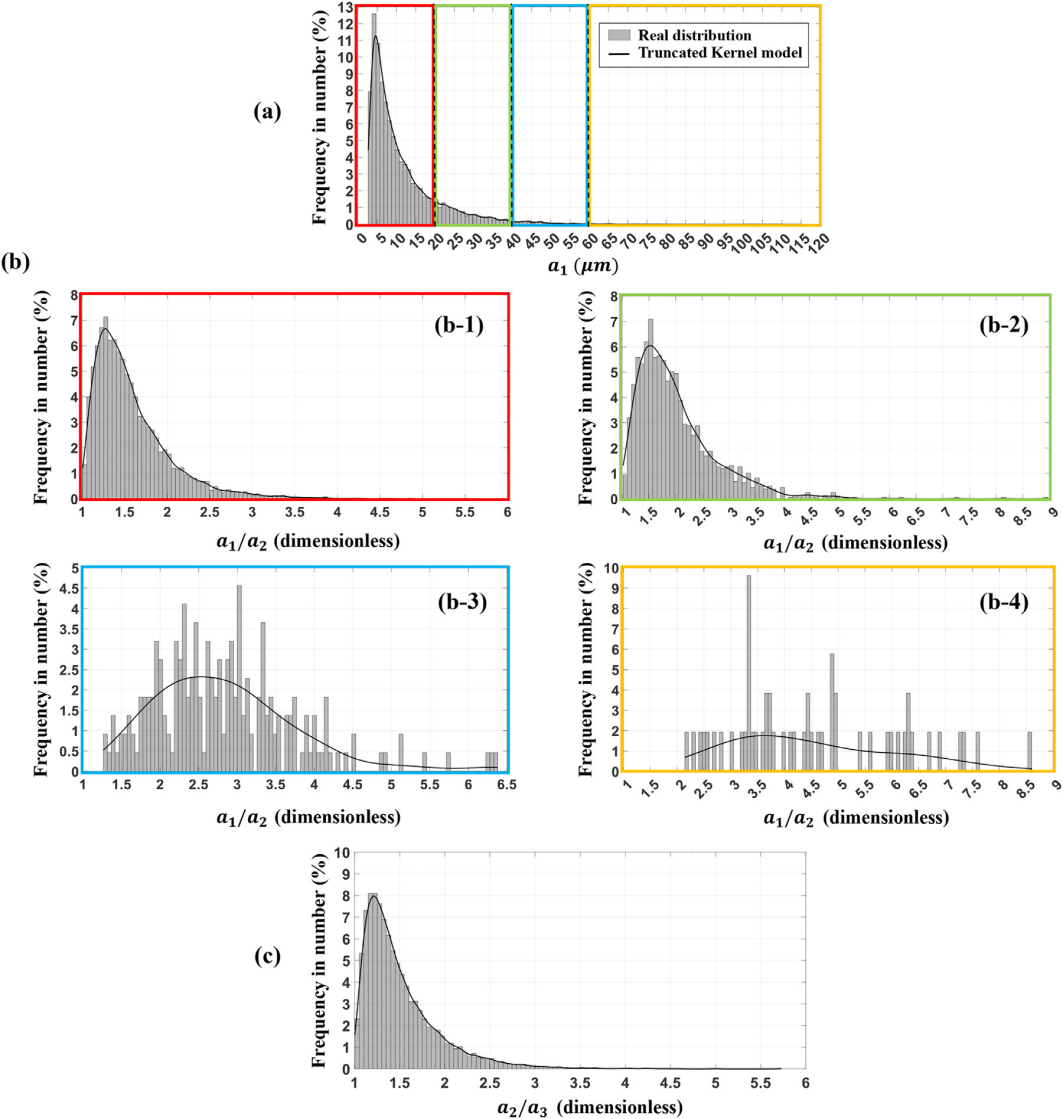
Comparison between the experimental distribution and the fitted truncated Kernel distribution for (a) a_1_ and (c) a_2_/a_3_ obtained from 12,812 particles. Comparison between experimental distribution and the fitted truncated Kernel distribution for a1/a2 in the zone a_1_≤20 µm (b-1), 20<a_1_≤40 µm (b-2),  40<_a1_ ≤60 µm (b-3) and a_1_>60 µm (b-4) obtained from 10,945, 1596, 219 and 52 particles, respectively.

Fig. 4 shows the real correlations between the axes a_1_, a_2_ and a3, for the 12,812 particles (grey bullets) analysed from the 12 cuboid samples extracted from the tomographic image. The red bullets present an example of generated axes values for 15,000 particles when a_1_/a_2_ are generated according to a single Kernel distribution defined on the entire a_1_ zone while the blue bullets present an example of generated axes values for 15,000 particles where a_1_/a_2_ is generated according to four Kernel distributions defined in four separate zones of a_1_. The experimental correlations between a_1_, a_2_ and a_3_ (grey bullets) are better described in the case where a_1_/a_2_ is generated in four separate zones of a_1_ (blue bullets) contrary to the case where a_1_/a_2_ is generated on the entire a_1_ zone (red bullets). As shown in Fig. 4*,* the preservation of the correlation between a_1_ & a_2_ (column a) and a_2_ & a_3_ (column c) tends to also preserve the correlation between a_1_ & a_3_ (column b). Fig. 5 presents the comparison between the experimental distribution and the fitted truncated Kernel distribution for the axis a_1_ (a) and a_2_/a_3_ (c). The same comparison is performed for a_1_/a_2_ (b) in the zone a_1_ ≤ 20 µm (b-1); 20 < a_1_ ≤ 40 µm (b-2); 40 < a_1_ ≤ 60 µm (b-3) and a_1_ > 60 µm (b-4).

**Fig. 6:**
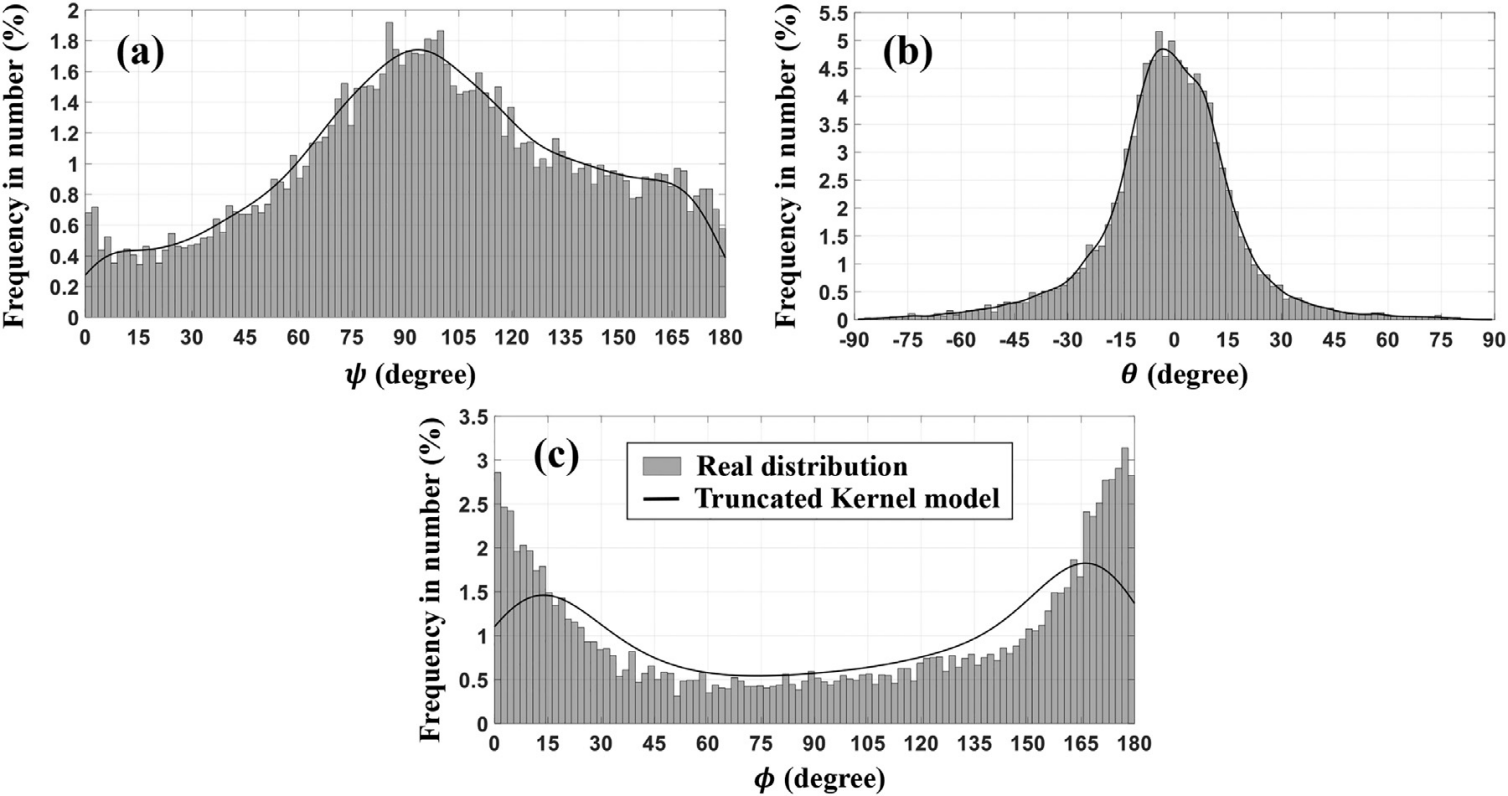
Comparison between experimental distribution and the fitted truncated Kernel distribution for (a) roll angle ψ (b) elevation angle θ and (c) azimuth angle ϕ obtained from 12,812 particles.

The Euler angles (ψ,θ,ϕ) were randomly generated according to the truncated Kernel distribution fitted to their real distribution as shown in Fig. 6.

For other simulation needs, the experimental distribution of the diameter d of the equivalent spheres, in volume, of the real particles could be also fitted using the truncated Kernel law (see Fig. S3.1 of suppl. material).

**Step 3**. Based on the Kernel distribution previously identified, about 10 (3D) microstructures $(50\;\times \;50\;\times \;100\;\mu m^{3})\;$of the composite Polypropylene/cellulose containing size-heterogeneous ellipsoidal particles with $\varphi_{p}=2.96\%$ were generated using the procedure described in Figs. 1 and 2. Higher volume fraction (up to 19.91%) could be also generated without too much impact on computation time (Fig. 4).

Structures with size-heterogeneous spherical particles could be also generated (Fig. S3.2 of suppl. material).

## Funding

This project has received funding from the European Union's Horizon 2020 research and innovation program under grant agreement No 773375. 3SR is part of the labex Tec 21 (ANR-11-LBX-0030) and of Institute Carnot Polynat (ANR-16-CARN-0025–01).

## Declaration of Competing Interest

The authors declare that they have no known competing financial interests or personal relationships that could have appeared to influence the work reported in this paper.

## Data Availability

I have shared the link toward raw data via DOI (see in the manuscript)

## Appendix Supplementary materials

Image, application 1
