# Supplementary material for: Cascading (3D) reconstruction procedure of composite structures from microtomography data
Source: MethodsX. 2023 Apr 9;10:102177. doi: 10.1016/j.mex.2023.102177 (PMC10130495; doi:10.1016/j.mex.2023.102177)
Supplement: Supplementary file 1 [file mmc1.docx]

## Supplementary material

### **S1. Dimension of cuboid samples extracted from the global (3D) tomographic image:**

**Table S1.1:**  Dimension of twelve small cuboid samples of same high of 250 µm extracted from different zones of the global (3D) tomographic image of real dimension of 1844×1403×298 µm^3^

| **Sample** | **Dimension**  **(length x depth x height) µm^3^** |
| --- | --- |
| **1** | 500 × 245 × 250 |
| **2** | 500 × 269 × 250 |
| **3** | 500 × 217 × 250 |
| **4** | 500 × 202 × 250 |
| **5** | 500 × 266 × 250 |
| **6** | 500 × 181 × 250 |
| **7** | 500 × 205 × 250 |
| **8** | 500 × 117 × 250 |
| **9** | 500 × 165 × 250 |
| **10** | 500 × 98 × 250 |
| **11** | 500 × 133 × 250 |
| **12** | 500 × 194 × 250 |

### **S2. Conversion expressions of Tait-Bryan angles into Euler angles:**

**The Tait-Bryan angles** $(\psi,\theta,\phi)$ collected from **ImageJ (MorphoLibj plugin)** represent the rotation of ellipsoidal particle as principal rotations about the **space-fixed principal axes** (extrinsic rotations), namely the x, y and z axes (x-y-z rotation convention). The first rotation is by roll angle $\psi$ about the x-axis. The second is by elevation angle $\theta$ about the y-axis. The third is by azimuth angle $\phi$ about the z-axis. These three successive rotations around the original axes are represented by multiplying the three elementary rotation matrices $R_{(\psi,\theta, \phi)}=R_{z}(\phi)\cdot R_{y}(\theta)\cdot R_{x}(\psi)$ in this order. The obtained global rotation matrix is expressed as follows:

$R_{(\psi,\theta, \phi)}=\left[ \begin{matrix} cos\theta\cdot cos\phi& sin\psi\cdot sin\theta\cdot cos\phi-cos\psi\cdot sin\phi& cos\psi\cdot sin\theta\cdot cos\phi+sin\psi\cdot sin\phi\\ cos\theta\cdot sin\phi& sin\psi\cdot sin\theta\cdot sin\phi+cos\psi\cdot cos\phi& cos\psi\cdot sin\theta\cdot sin\phi-sin\psi\cdot cos\phi\\ -sin\theta& sin\psi\cdot cos\theta& cos\psi\cdot cos\theta\end{matrix} \right]$Eq. S2.1

For subsequent needs, the matrix Eq. A1 can be represented as follows:

$R_{(\psi,\theta, \phi)}=\left[ \begin{matrix} m_{11} & m_{12} & m_{13} \\ m_{21} & m_{22} & m_{23} \\ m_{31} & m_{32} & m_{33} \end{matrix} \right]$Eq. S2.2

It should be noted that the variation intervals of the angles $\psi,\theta$and $\phi$ used by MorphoLibJ plugin are $[-180^{\circ},180^{\circ}]$, $[-90^{\circ},90^{\circ}]$ and $[-180^{\circ},180^{\circ}]$ respectively.

**The classical Euler angles** $(\gamma,\beta, \alpha)$ used in **Comsol** represent the rotation of ellipsoidal particle as principal rotations about the **space-fixed principal axes** (extrinsic rotations), namely the x and z axes (z-x-z rotation convention). The first rotation is by intrinsic angle $\gamma$ about the z-axis. The second is by nutation angle $\beta$ about the x-axis. The third is by precession angle $\alpha$ about the z-axis. These three successive rotations around the original axes are represented by multiplying the three elementary rotation matrices $R_{(\gamma,\beta, \alpha)}= R_{z}(\alpha)\cdot R_{x}(\beta)\cdot R_{z}(\gamma)$ in this order. The obtained global rotation matrix is expressed as follows:

$R_{(\gamma,\beta, \alpha)}=\left[ \begin{matrix} -sin\gamma\cdot cos\beta\cdot sin\alpha+cos\gamma\cdot cos\alpha& -cos\gamma\cdot cos\beta\cdot sin\alpha-sin\gamma\cdot cos\alpha& sin\beta\cdot sin\alpha\\ sin\gamma\cdot cos\beta\cdot cos\alpha+cos\gamma\cdot sin\alpha& cos\gamma\cdot cos\beta\cdot cos\alpha-sin\gamma\cdot sin\alpha& -sin\beta\cdot cos\alpha\\ sin\gamma\cdot sin\beta& cos\gamma\cdot sin\beta& cos\beta\end{matrix} \right]$Eq. S2.3

It should be noted that the variation intervals of the angles $\gamma,\beta$and $\alpha$ used in Comsol are $[-180^{\circ},180^{\circ}]$, $[-90^{\circ},90^{\circ}]$ and $[-180^{\circ},180^{\circ}]$ respectively.

Matching the elements of the matrix $R_{(\gamma,\beta, \alpha)}$ (Eq. A3) to those of $R_{(\psi,\theta, \phi)}$ (Eq. A2) that are known, the classical Euler angles $(\gamma,\beta,\alpha)$ are identified (see also Table 1 in page 19 of the reference of Berner 2008):

If $m_{33}\neq\pm1$:

$\left\{ \begin{aligned} \gamma=arctan2(m_{31},m_{32}) \\ \beta=arccos(m_{33}) \\ \alpha=arctan2(m_{13},{-m}_{23}) \end{aligned} \right.$ Eq. S2.4

If $m_{33}=1$:

$\left\{ \begin{aligned} any values of \gamma\\ \beta=0 \\ \alpha=arctan2\left( m_{21},m_{11} \right)- \gamma\end{aligned} \right.$ Eq. S2.5

If $m_{33}=-1$:

$\left\{ \begin{aligned} any values of \gamma\\ \beta=\pi\\ \alpha=arctan2\left( m_{21},m_{11} \right)+ \gamma\end{aligned} \right.$Eq. S2.6

where arctan2(y,x) is the arc tangent function of the ratio between the two variables y and x that extends the arc tangent function to the all four quadrants. The function arctan2 is available in most programming languages. For more detail about this conversion, see the reference of Berner (2008).

### **S3. Reconstruction of (3D) structures with size-heterogeneous spherical particles:**

***Figure S3.1:*** *Comparison between experimental distribution and the fitted truncated Kernel distribution for diameter d (corresponding to spheres volumes equal to real particles volumes) obtained from 12812 particles.*

**Figure S3.2:**  Representation of the RVE showing generated structures with size-heterogeneous spherical particles: (a) bi-phasic structures and (b) tri-phasic structures of interphase thickness of 2µm (*purple color*), both generated using the algorithm proposed in that work. The structures are *generated for particles volume fraction corresponding to true composite materials (*$\varphi_{p}=2.96-6.06-12.67-19.91 \%v/v$*).*
